# Lipid metabolism-MAFLD crosstalk: mechanisms and therapy

**DOI:** 10.3389/fendo.2026.1785178

**Published:** 2026-03-18

**Authors:** Bojia Li, Shengai Piao, Yin Fu, Qiang Fu, Peiyao Qin, Weitai Kong, Yidi Ma, Zhe Zhang, Xue Fang, Xiaoyang Hu

**Affiliations:** 1 Basic Medical College, Heilongjiang University of Chinese Medicine, Harbin, Heilongjiang, China; 2 Department of Pediatrics, The Second Affiliated Hospital of Heilongjiang University of Chinese Medicine, Harbin, Heilongjiang, China; 3 Heilongjiang University of Chinese Medicine, Harbin, Heilongjiang, China

**Keywords:** lipid metabolism, lipotoxicity, mechanisms, metabolic dysfunction-associated fatty liver disease, therapeutic perspectives

## Abstract

Metabolic dysfunction-associated fatty liver disease (MAFLD) has become the most prevalent chronic liver disorder worldwide, encompassing a spectrum that ranges from simple steatosis to metabolic dysfunction-associated steatohepatitis (MASH) and hepatic fibrosis. However, its precise pathogenic mechanisms remain incompletely understood, and effective, specific pharmacological treatments are still lacking. Disruption of hepatic lipid metabolic homeostasis represents a central event in the onset and progression of MAFLD. With advances in lipidomics and metabolomics, researchers can now more accurately delineate the aberrant accumulation of specific lipid species within hepatocytes and their pivotal roles in triggering insulin resistance, oxidative stress, and inflammatory responses. This review systematically summarizes the core mechanisms by which hepatic lipid metabolic dysregulation drives MAFLD progression and highlights recent advances in therapeutic strategies targeting lipotoxic pathways, metabolic reprogramming, and related molecular targets. These insights aim to provide a theoretical basis and new perspectives for future research and clinical intervention in this field.

## Graphical Abstract

**Figure:**
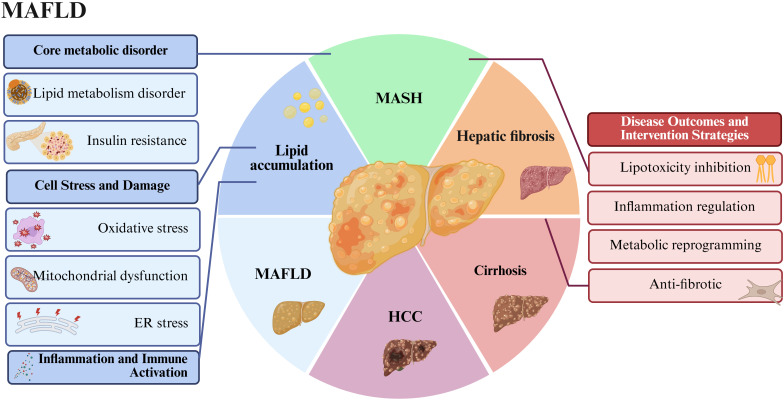
This graphical abstract shows MAFLD progression (steatosis to HCC), highlights hepatocyte lipid accumulation and driving mechanisms, outlines interventions, and synthesizes the review’s key pathogenic and therapeutic links.

## 1 Introduction

Metabolic dysfunction-associated fatty liver disease (MAFLD), formerly known as non-alcoholic fatty liver disease (NAFLD), is the most prevalent chronic liver condition worldwide, affecting an estimated 25% of the adult population (1, 2). The disease encompasses a progressive spectrum from simple hepatic steatosis to non-alcoholic steatohepatitis (NASH), which can advance to cirrhosis and hepatocellular carcinoma (HCC) (3, 4). In 2020, an international panel of experts proposed the term MAFLD to better reflect its strong etiological links with metabolic dysfunctions such as obesity, type 2 diabetes mellitus, and hypertriglyceridemia, moving away from the previous emphasis on excluding alcohol use. This redefinition underscores that MAFLD is not an isolated hepatic disorder but rather a multisystem disease intricately connected with cardiovascular and metabolic comorbidities A substantial proportion of patients present with metabolic syndrome, which markedly increases their risk of cardiovascular disease and extrahepatic malignancies (5, 6). These clinical associations compel deeper investigation into the underlying pathogenic mechanisms to develop more effective therapeutic strategies and mitigate the growing global health burden.

As the central organ of systemic lipid metabolism, the liver plays a pivotal role in maintaining lipid homeostasis. Disruption of this balance is widely regarded as the “first hit” in the pathogenesis of MAFLD (7, 8). Although this concept is well recognized, the precise mechanistic links between lipid metabolic disturbances and MAFLD onset and progression remain to be fully elucidated. In this review, we summarize recent advances in this field, with particular emphasis on the critical roles of specific lipid species in MAFLD pathophysiology (9, 10).

Unlike traditional perspectives that regard lipids merely as energy storage substrates, emerging evidence highlights that distinct lipid metabolites function as signaling molecules that not only mediate insulin resistance (IR) but also act as central messengers linking metabolic dysregulation to hepatocellular injury, inflammation, and fibrosis (11, 12). By systematically elucidating how these toxic lipids impair insulin signaling, activate inflammasomes, induce mitochondrial and endoplasmic reticulum stress, and promote hepatic stellate cell activation, this review provides a conceptual framework for the development of innovative therapeutic strategies targeting MAFLD (13, 14).

Building on this paradigm shift, recent studies have further underscored that hepatic lipid metabolism is governed by a sophisticated regulatory network, rather than isolated metabolic pathways (15). Dysregulation of this integrated network, rather than the dysfunction of individual nodes, drives the pathological accumulation of specific toxic lipid species such as ceramides, diacylglycerols, and free cholesterol, which collectively propagate insulin resistance, oxidative stress, and inflammatory cascades in MAFLD. This network-based view shifts the therapeutic paradigm from simply modulating individual metabolic pathways toward selectively targeting these pathogenic lipid species and restoring overall network balance. Consequently, identifying strategies that selectively neutralize toxic lipids while preserving physiological lipid functions has emerged as a promising direction for MAFLD drug development, offering opportunities for multi-target interventions that address the complex and interconnected nature of the disease.

## 2 Physiological homeostasis of hepatic lipid metabolism

### 2.1 Physiological roles of hepatic lipids

As the central organ of systemic lipid metabolism, the liver plays a fundamental role in maintaining whole-body lipid homeostasis through precise regulation of key metabolic pathways. It is responsible for both endogenous lipid synthesis and lipid export (16, 17). Lipids serve several essential physiological functions within the metabolic network: (i) Energy storage: Triglycerides (TGs) act as the principal energy reservoirs, stored in the liver and adipose tissue to provide efficient energy substrates during fasting or periods of high energy demand (18, 19). (ii) Structural components: Lipids constitute the fundamental scaffolding of biological membranes, maintaining membrane fluidity, barrier function, and the structural integrity of organelles such as mitochondria and the endoplasmic reticulum (18, 20, 21). (iii) Metabolic signaling molecules: Lipid metabolites serve as key regulatory mediators that influence insulin sensitivity and inflammatory responses, acting as crucial hubs that connect local and systemic metabolic homeostasis (18, 22). Therefore, chronic dysregulation of hepatic lipid metabolism disrupts lipid homeostasis, leading to abnormal hepatic lipid accumulation and initiating the onset of MAFLD.

### 2.2 Lipids in the metabolic system

**Figure 1:**
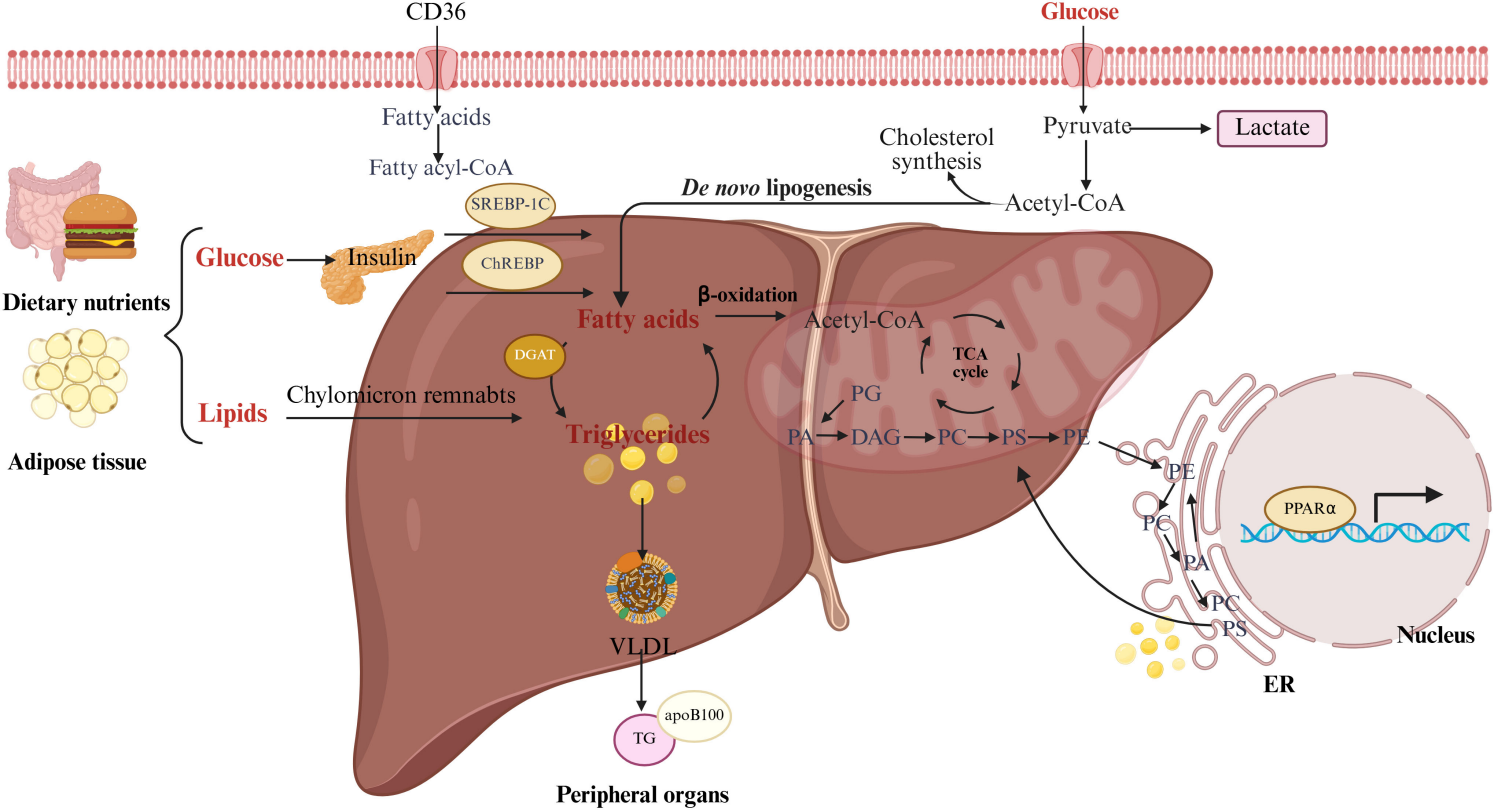
Major processes of lipid metabolism: uptake, synthesis, and utilization. Schematic overview of hepatic lipid metabolic fluxes following nutrient intake. Glucose is converted to fatty acids via insulin-regulated *de novo* lipogenesis (DNL). Dietary lipids are taken up as chylomicron remnants mainly via CD36, a fatty acid translocase. Fatty acids are either oxidized in mitochondria to produce acetyl-CoA for the TCA cycle, cholesterol biosynthesis and histone acetylation, or esterified via DGAT into diacylglycerols (DAGs) and triglycerides (TGs) for storage in lipid droplets. Excess fatty acids also generate membrane phospholipids, including phosphatidic acid (PA), phosphatidylglycerol (PG), phosphatidylcholine (PC), phosphatidylethanolamine (PE), and phosphatidylserine (PS). TGs are assembled with apoB100 into VLDLs for secretion to peripheral tissues. PA acts as a central phospholipid intermediate, and PG is a key precursor for cardiolipin, supporting membrane structure and signaling.

Major organs involved in lipid metabolism include the liver, adipose tissue, intestine, and skeletal muscle. At the subcellular level, the process engages the endoplasmic reticulum, and mitochondria, while lipoproteins mediate inter-organ lipid transport (23) (**Figure 1**).

#### 2.2.1 Sources of lipids

Hepatic fatty acids are primarily derived from three sources, with their relative contributions dynamically regulated according to nutritional status to maintain metabolic balance (24). Lipids from the diet are digested and absorbed in the intestine, then transported into circulation as chylomicrons. Hydrolysis of chylomicron triglycerides releases free fatty acids (FFAs), which are taken up by hepatocytes via specific transport proteins (25). During fasting or increased energy demand, adipose tissue lipolysis is activated by hormone-sensitive enzymes, releasing FFAs and glycerol, the latter serving as a gluconeogenic precursor (24, 26). Hepatic fatty acid synthesis from carbohydrates is stimulated under conditions of carbohydrate excess, regulated by transcription factors SREBP-1c and ChREBP. Although it accounts for only about 5% of hepatic fatty acids under normal conditions, its contribution markedly increases during metabolic disorders (27, 28).

#### 2.2.2 Intracellular processing of fatty acids in hepatocytes

Hepatocytes process fatty acids primarily through two pathways: oxidative metabolism and esterification. β-oxidation in mitochondria is the principal route for fatty acid degradation, with its rate-limiting step catalyzed by CPT1A. This process produces acetyl-CoA, which can either enter the tricarboxylic acid cycle for ATP generation or be converted into ketone bodies to supply energy to peripheral tissues (29, 30). The transcription factor PPARα acts as the central regulator of this pathway (24, 31). When fatty acid supply exceeds oxidative capacity, the surplus is converted into triglycerides through the esterification pathway, catalyzed by key enzymes such as DGAT. The resulting triglycerides are stored in lipid droplets or utilized for lipoprotein assembly (24). The dynamic balance between these two processes is crucial for maintaining hepatic lipid homeostasis (32).

#### 2.2.3 Hepatic lipid export

The liver prevents intracellular lipid overload through multiple export mechanisms. Very-low-density lipoprotein (VLDL) secretion is the primary pathway, involving the initial assembly of ApoB100 with lipids in the endoplasmic reticulum and subsequent maturation in the Golgi apparatus, tightly regulated by various factors (33, 34). Additional export routes include ketone body production and biliary secretion, which act in concert under different physiological conditions to maintain hepatic lipid balance (35).

## 3 Pathogenesis of MAFLD

**Figure 2:**
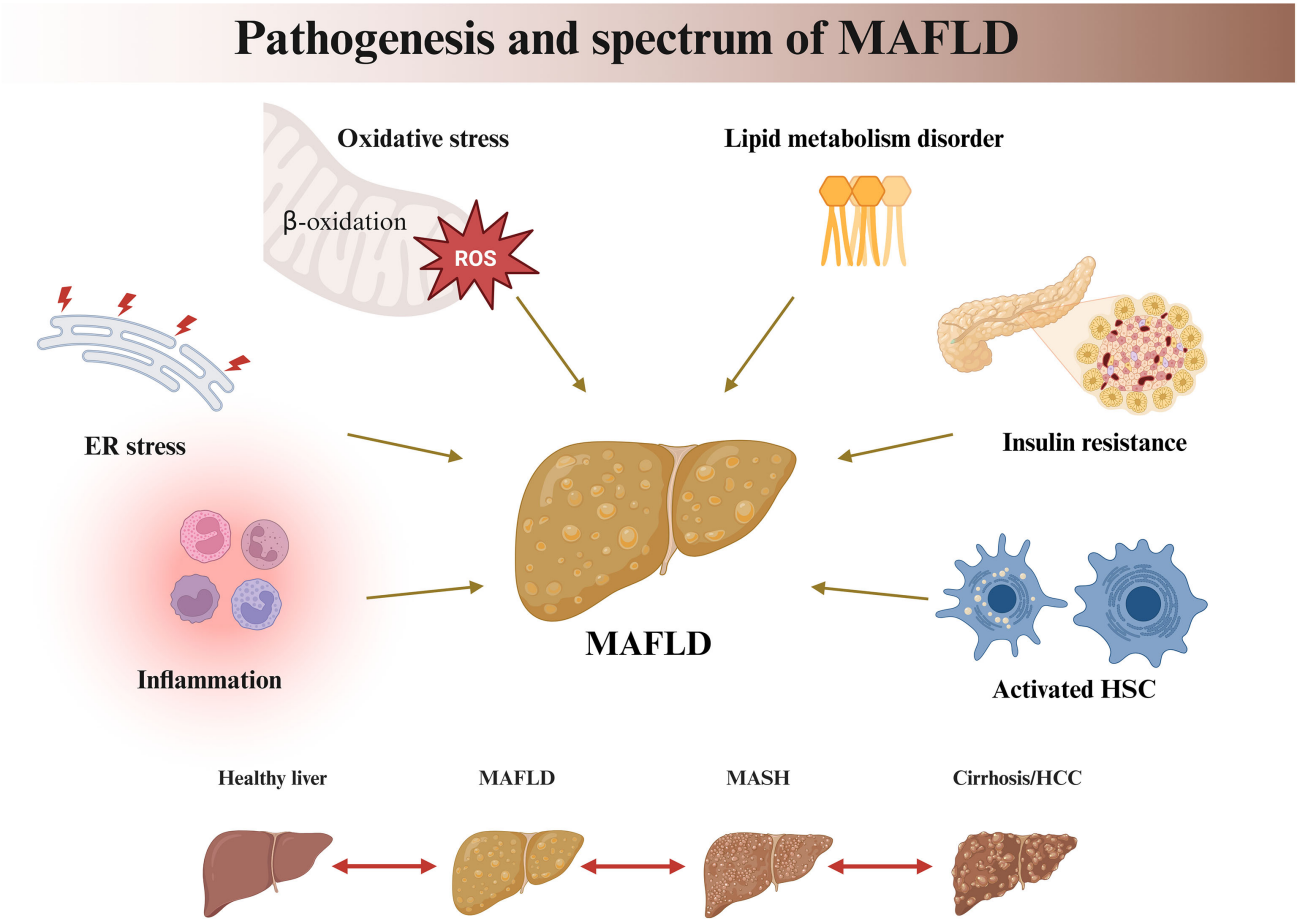
Pathogenesis and spectrum of MAFLD. Integrated mechanisms driving MAFLD development and progression, highlighting endoplasmic reticulum (ER) stress, oxidative stress, lipid metabolic dysregulation, insulin resistance, inflammation, and hepatic stellate cell (HSC) activation. The disease spectrum is depicted from a healthy liver to MAFLD and MASH, with potential progression to cirrhosis and hepatocellular carcinoma (HCC).

The pathogenesis of MASLD is fundamentally rooted in systemic metabolic dysfunction. Insulin resistance in adipose tissue, skeletal muscle, and the liver itself creates a permissive environment for hepatic lipid accumulation, while hyperinsulinemia and hyperglycemia directly promote *de novo* lipogenesis. Thus, MASLD can be viewed as the hepatic component of the metabolic syndrome, with its progression driven by the interplay between systemic metabolic stress and local hepatic responses. Although this metabolic framework is now widely accepted, the precise molecular mechanisms linking systemic dysfunction to hepatocellular injury remain incompletely understood. Current evidence implicates several interrelated pathophysiological processes (**Figure 2**). Disruption of hepatic lipid metabolic homeostasis, together with increased mobilization of endogenous free fatty acids (FFAs) from adipose tissue and excessive uptake of exogenous FFAs, collectively imposes a dual metabolic burden that drives the accumulation of triglyceride precursors (36, 37). Concurrently, impaired mitochondrial β-oxidation and defective very-low-density lipoprotein (VLDL) assembly or secretion reduce hepatic lipid clearance efficiency, ultimately resulting in pathological triglyceride deposition within hepatocytes (38–40).

When hepatocellular TG storage capacity or compensatory oxidative metabolism is exceeded, excessive FFAs and reactive oxygen species (ROS) synergistically induce lipid peroxidation and oxidative stress, triggering endoplasmic reticulum (ER) stress and a cascade of lipotoxic reactions (41, 42). Importantly, lipotoxicity does not arise from the accumulation of neutral TGs but rather from the selective enrichment of toxic lipid species such as ceramides and diacylglycerols (DAGs) (43, 44). Clinical studies have confirmed that hepatic levels of these lipotoxic species are positively correlated with the severity of liver injury at the non-alcoholic steatohepatitis (MASH) stage (45). Subsequently, IR and lipotoxicity form a vicious cycle that further disrupts lipid metabolic homeostasis (46, 47). Lipotoxicity-induced organelle dysfunction and chronic inflammation drive the activation of hepatic stellate cells (HSCs) and promote excessive extracellular matrix deposition, ultimately leading to hepatic fibrosis (39, 48). Notably, during the progression of MAFLD toward end-stage liver diseases such as cirrhosis and HCC, alterations in ceramide subspecies composition and abnormalities in the hepatic phosphatidylcholine (PC) to phosphatidylethanolamine (PE) ratio (PC/PE) further emphasize the critical role of lipid metabolic homeostasis in MAFLD pathogenesis.

## 4 Lipid metabolism disorders and regulatory mechanisms in MAFLD

### 4.1 Lipid alterations in liver diseases associated with the development of MAFLD

Over the past decades, extensive metabolomic analyses in both MAFLD patients and animal models have revealed significant alterations in lipid metabolite profiles. These studies suggest that lipid metabolites may play crucial roles in the onset and progression of MAFLD, with hepatic lipid metabolism-related molecules displaying abnormal regulation. Although the precise mechanisms by which specific lipids contribute to MAFLD remain incompletely understood, increasing evidence from genetic, epidemiological, and biochemical research underscores the central importance of lipid homeostasis in MAFLD pathogenesis. (**Table 1**).

**Table 1:** Lipidomic and metabolite changes associated with MAFLD progression.

Metabolite name	Lipid Category	Main finding	Ref.
TG	Glycerides	TG serves as the main form of lipid storage in hepatocytes and is markedly elevated during MAFLD progression	(48–50)
DAG	Glycerides	DAG aggravates hepatic insulin resistance by activating PKCϵ, thereby driving inflammation and disease progression	(51–53)
PC/PE	Phospholipids	A decreased PC/PE ratio disrupts membrane stability, enhances TG accumulation, and increases hepatocyte susceptibility to injury; other phospholipids promote MAFLD progression by impairing insulin signaling, triggering inflammation, and inducing apoptosis	(54–59)
FC	Sterol Lipids	FC abnormally accumulates in mitochondrial membranes, disrupting the electron transport chain and inducing oxidative stress; it also activates hepatic stellate cells and Kupffer cells, promoting persistent fibrotic and inflammatory signaling	(56, 60–64)
Cer	Sphingolipids (SLs)	Abnormal accumulation of Cer drives the transition from MAFLD to MASH, elevated C16:0-Cer in the *de novo* synthesis pathway induces hepatocellular injury, whereas inhibition of Cer synthesis alleviates steatosis and improves insulin sensitivity	(65)
SM	Sphingolipids (SLs)	SM, hydrolyzed by sphingomyelinases to generate Cer, decreases during MAFLD progression, disrupting lipid raft structure, impairing signal transduction, enhancing hepatocyte inflammatory sensitivity, and accelerating the conversion from MAFLD to MASH	(54, 66, 67)

#### 4.1.1 Glycerolipids

In the study of MAFLD, elevated levels of polyunsaturated TG have been shown to induce hepatic steatosis and cause hepatocellular injury (49). However, targeted interventions in both MAFLD patients and mouse models can effectively attenuate the degree of steatosis (50, 51). In obese individuals, approximately 60% of hepatic TG originates from FFA released through adipose tissue lipolysis. In patients with hypertriglyceridemia and hyperinsulinemia, hepatic *de novo* lipogenesis (DNL) is markedly upregulated during fasting, serving as a major source of TG synthesis (52). Moreover, DAG levels are significantly elevated in MAFLD and contribute to hepatic insulin resistance by activating PKCϵ, which interferes with insulin signaling. In choline-deficient, high-fat diet-induced MASH mouse models, hepatic DAG accumulation directly drives inflammatory injury and promotes disease progression from simple steatosis to MASH (53–55).

#### 4.1.2 Phospholipids

Phospholipids are essential structural components of biological membranes, playing critical roles in maintaining membrane integrity, regulating lipid transport, and mediating signal transduction. In MAFLD, the hepatic PC/PE ratio is often significantly reduced (56). Lipidomic analyses have demonstrated that all PE subspecies are markedly elevated in MASH mouse models. PC synthesis depends on the PE methylation pathway mediated by phosphatidylethanolamine N-methyltransferase (PEMT), and downregulation of key genes in this pathway leads to decreased PC production, thereby exacerbating hepatic TG accumulation (57). A reduced PC/PE ratio destabilizes membrane structure and increases hepatocyte susceptibility to secondary insults (56, 58, 59). In addition, other phospholipids, such as lysophosphatidylcholine (LysoPC), phosphatidylinositol (PI) derivatives, phosphatidic acid (PA), and phosphatidylserine (PS), also contribute to MAFLD progression by disrupting insulin signaling, promoting inflammation, and inducing apoptosis, collectively driving the transition from MAFLD to MASH and fibrosis (60).

#### 4.1.3 Free cholesterol

Lipidomic studies of human liver tissues have revealed that free cholesterol (FC) levels are elevated in MASH compared with simple steatosis, whereas esterified cholesterol remains unchanged (61). FC serves as a key discriminative metabolite between simple steatosis and MASH (62, 63). Excess FC accumulates abnormally within mitochondrial membranes, directly impairing the electron transport chain and inducing oxidative stress (64, 68). Moreover, FC can activate hepatic stellate cells and Kupffer cells, thereby promoting persistent activation of fibrotic and inflammatory signaling pathways (69).

#### 4.1.4 Sphingolipids

Sphingolipids (SLs) are fundamental structural and signaling components of eukaryotic cell membranes, and their dysregulation plays a pivotal role in MAFLD progression. Among them, ceramides (Cer) are recognized as key drivers of disease transition from MAFLD to MASH (65, 66). Elevated hepatic Cer levels arise mainly through two pathways. First, *de novo* synthesis in the ER, catalyzed sequentially by serine palmitoyltransferase (SPT) and ceramide synthases (CerS). Studies show that deletion of CerS2 leads to compensatory elevation of hepatic C16:0-Cer, which induces hepatocyte death and abnormal proliferation, whereas pharmacological inhibition of Cer synthesis alleviates steatosis and restores insulin sensitivity in mice (67). Secondly, hydrolysis of sphingomyelin (SM) by sphingomyelinases such as acid sphingomyelinase (ASMase). SM is a major component of lipid rafts, and downregulation of sphingomyelin synthase 1 (SMS1), which catalyzes the conversion of Cer to SM, exacerbates lipotoxicity and promotes hepatocyte pyroptosis (54). During MAFLD progression, declining SM levels disrupt lipid raft structure, compromise membrane integrity, and impair key signaling pathways. These changes heighten hepatocyte sensitivity to proinflammatory cytokines and accelerate the transition from simple steatosis to MASH (70, 71).

Lipotoxicity, rather than simple triglyceride accumulation, is increasingly recognized as a central driver of disease progression in MAFLD, as distinct toxic lipid species actively induce cellular stress responses, inflammation, and regulated hepatocyte death pathways (72–74). Saturated free fatty acids, ceramides, diacylglycerols, and free cholesterol function as bioactive mediators that disrupt endoplasmic reticulum and mitochondrial homeostasis, leading to unfolded protein response activation, excessive reactive oxygen species production, and apoptotic signaling in hepatocytes (75–77). Beyond direct cytotoxicity, lipotoxic stress engages innate immune pathways that amplify hepatic inflammation. Recent studies demonstrate that mitochondrial dysfunction, oxidative stress, and lysosomal destabilization collectively promote activation of the NLRP3 inflammasome in metabolic dysfunction–associated steatotic liver disease, resulting in caspase-1–dependent maturation of IL-1β and IL-18 and contributing to hepatocellular injury and disease progression (78–80). These findings support the concept that therapeutic strategies should prioritize selective neutralization of toxic lipid intermediates rather than indiscriminate reduction of total hepatic lipid content.

### 4.2 Regulatory mechanisms of lipid remodeling

#### 4.2.1 SREBP-1c: a central regulator of fatty acid and cholesterol synthesis

SREBP-1c is a key transcription factor controlling the DNL pathway and plays a pivotal role in the pathogenesis of MAFLD (81). Its expression and activity are tightly regulated by nutritional and hormonal cues, insulin and hyperglycemia promote SREBP-1c transcription and proteolytic activation, whereas polyunsaturated fatty acids exert inhibitory effects (82). Synthesized in the ER, SREBP-1c forms a complex with SREBP cleavage-activating protein (SCAP) and insulin-induced gene (INSIG). Notably, liver-specific deletion of SCAP alleviates steatosis but aggravates hepatic injury and fibrosis (58). Studies have shown that glycerol kinase expression positively correlates with SREBP-1c levels, and its knockdown markedly suppresses SREBP-1c and downstream lipogenic gene expression (83). Moreover, SREBP-1c regulates the autophagy process via the miR-216a-cystathionine-γ-lyase (CSE)-ULK1 sulfhydration axis (84). From a therapeutic standpoint, berberine inhibits SREBP-1c transcriptional activity by activating AMPK-mediated phosphorylation, thereby downregulating target genes such as SCD1. Experimental data confirm that SCD1 knockdown mimics berberine’s lipid-lowering effects, whereas SCD1 overexpression attenuates them (85). Furthermore, the interaction between the gut microbiota and the SREBP-1c/SCD1 signaling pathway has emerged as a promising therapeutic target for MAFLD (86) (**Table 2**).

**Table 2:** Key transcriptional regulators and signaling pathways governing hepatic lipid metabolism in MAFLD progression.

Regulator	Upstream regulators	Downstream targets	Function	Ref.
SREBP-1c	AMPK miR-216a	DGAT2, FASN, SCD1	SSREBP-1c is activated, upregulating FAS, SCD1, and DGAT1/2 expression, which significantly enhances *de novo* lipogenesis (DNL), elevates serum lipid levels, and promotes hepatic TG deposition.	(82, 83)
ChREBP	PKCα,Tcf7l2, Nogo	ACC, FAS, SCD1, LPK	Activation of ACC, FAS, SCD1, and LPK and other lipogenic genes leads to abnormal lipid synthesis and TG accumulation. It also activates the ER stress pathway, inducing insulin resistance.	(85–88)
PPARα	RAN, CRM1, AMPK	CPT1α, ACOX1	Upregulates ACOX1 to enhance peroxisomal β-oxidation, sustained activation of which can lead to endoplasmic reticulum stress and mitochondrial dysfunction, promoting lipid accumulation.	(89, 90)
FXR	PPARα	SHP-1, FGF15/19, PI3K/AKT/GSK3	Activation inhibits DNL and lipoprotein export in MASH mouse models, improving steatosis and reducing inflammation and fibrosis; it also upregulates activated PPARα fatty acid oxidation pathways and enhances insulin sensitivity via the PI3K/AKT/GSK3β signaling pathway.	(91–94)

#### 4.2.2 ChREBP: a critical bridge linking carbohydrate metabolism and lipid synthesis

ChREBP acts as a key metabolic regulator integrating glycolysis and lipogenesis. As a nutrient-sensing organ, the liver maintains lipid metabolic balance through ChREBP-mediated signaling networks. ChREBP expression is markedly induced during hepatic steatosis, and its overactivation upregulates lipogenic genes such as ACC, FAS, and SCD1, resulting in hepatic TG accumulation (87). ChREBP activity is precisely modulated at multiple levels. The PKCα signaling pathway specifically activates ChREBP transcriptional activity; overexpression of wild-type PKCα significantly enhances ChREBP and downstream target gene expression in primary hepatocytes, whereas dominant-negative PKCα has no such effect (88). The transcription factor TCF7L2 has been shown to inhibit the binding of the ChREBP/MLX complex to target gene promoters in an LXR-independent manner, revealing a novel mechanism for ChREBP regulation (95). Nutritional status also profoundly influences ChREBP activity. High-glucose and high-fructose diets strongly activate ChREBP and promote metabolic dysfunction. Nogo-B has been identified as a key mediator in this process, loss of Nogo or siRNA-mediated knockdown improves insulin sensitivity, activates the AMPK/PPARα pathway, and alleviates ER stress, collectively suppressing ChREBP activation and its downstream lipogenic gene expression (96). Thus, as a glucose-sensing transcription factor, ChREBP integrates nutritional signals and transcriptional networks to play an essential role in MAFLD development and progression (97).

#### 4.2.3 PPARα: the principal regulator of fatty acid oxidation

PPARα, a member of the nuclear receptor superfamily, is predominantly expressed in hepatocytes and regulates fatty acid transport and fatty acid oxidation (FAO) by activating the transcription of nuclear target genes (89). Substantial evidence indicates that PPARα expression is altered during the progression of MAFLD and MASH in both animal models and human patients (90). Hepatic PPARα levels are inversely correlated with the severity of steatosis, MASH, and fibrosis.

Preclinical studies have revealed that endogenous bile acid metabolites such as hyodeoxycholic acid (HDCA) exert anti-MAFLD effects through PPARα activation. Serum HDCA levels are significantly reduced in both patients and mouse models of MAFLD and negatively correlate with disease severity. Notably, hepatocyte-specific PPARα knockout abolishes the protective effects of HDCA, confirming the necessity of PPARα signaling in this process (98). Transcriptomic analyses have identified upregulation of acyl-CoA oxidase 1 (ACOX1), a key enzyme in the peroxisomal β-oxidation pathway regulated by PPARα, as a major node in hepatic lipid metabolism in humans, mice, and rats. Interestingly, recent studies have highlighted the dual role of PPARα activation: excessive activation of the PPARα-ACOX1 axis can lead to peroxisomal β-oxidation hyperactivation, oxidative stress, and mitochondrial damage, ultimately exacerbating hepatic lipid accumulation. These findings suggest that inappropriate activation of the PPARα pathway may also contribute to MAFLD pathogenesis (91).

#### 4.2.4 FXR: the cross-regulatory node of bile acid homeostasis and lipid metabolism

FXR, a bile acid-activated nuclear receptor predominantly expressed in hepatocytes and enterocytes, serves as a central regulator of bile acid homeostasis by controlling bile acid synthesis, secretion, and enterohepatic circulation. Upon activation by elevated bile acid levels, FXR initiates a negative feedback loop that suppresses key hepatic enzymes (e.g., CYP7A1) involved in bile acid synthesis (92). Beyond bile acid metabolism, FXR activation confers broad metabolic benefits relevant to MAFLD, including reductions in hepatic and plasma triglyceride levels, attenuation of inflammation, and enhancement of insulin sensitivity (93). In the liver, FXR activation induces the small heterodimer partner (SHP), which inhibits SREBP-1c transcriptional activity, thereby repressing *de novo* lipogenesis. In the intestine, FXR activation stimulates the release of fibroblast growth factor 19 (FGF19), which acts endocrinally on the liver to suppress bile acid synthesis and modulate lipid and glucose metabolism (94). These dual roles position FXR as a key integrator of hepatic and intestinal metabolic signals. Importantly, FXR activity is finely tuned by the gut microbiota through the production of secondary bile acids. For example, microbiota-derived hyodeoxycholic acid (HDCA) can antagonize intestinal FXR, leading to upregulation of the alternative bile acid synthesis enzyme CYP7B1 and concurrent activation of PPARα-mediated fatty acid oxidation, thereby alleviating hepatic steatosis (99). Moreover, FXR improves insulin sensitivity through the PI3K/AKT/GSK3β signaling cascade, promoting glycogen synthesis and glucose utilization (100). The intricate interplay between the gut microbiota, bile acid metabolism, and FXR signaling forms the molecular foundation of the gut–liver axis, which will be discussed in detail in Section 4.3.

### 4.3 The gut–liver axis: role of gut microbiota in hepatic lipid metabolism

FXR serves as a critical link between bile acid metabolism and hepatic lipid homeostasis, but it operates within the broader gut–liver axis, where the gut microbiota acts as a master modulator. This section expands the discussion to the systemic role of the gut microbiota in MAFLD. The gut microbiota shapes the bile acid pool via enzymatic conversion of primary to secondary bile acids (101–103), which exhibit distinct FXR activation potencies. Dysbiosis-associated bile acid alterations in MAFLD patients are closely linked to impaired FXR signaling and metabolic dysregulation (72, 101). Beyond bile acids, microbiota-derived short-chain fatty acids (SCFAs) activate GPR41/43, promoting insulin sensitivity and suppressing lipogenesis, while tryptophan metabolites modulate inflammation. The gut microbiota–bile acid–FXR axis also exerts immunomodulatory effects. Dysbiosis impairs intestinal barrier integrity, increasing systemic exposure to LPS and amplifying hepatic inflammation. Conversely, FXR negatively regulates NF-κB and NLRP3 inflammasome, protecting against inflammation-driven MAFLD progression (104–106).

### 4.4 Interplay of key transcriptional regulators: an integrated lipid metabolic network

**Figure 3:**
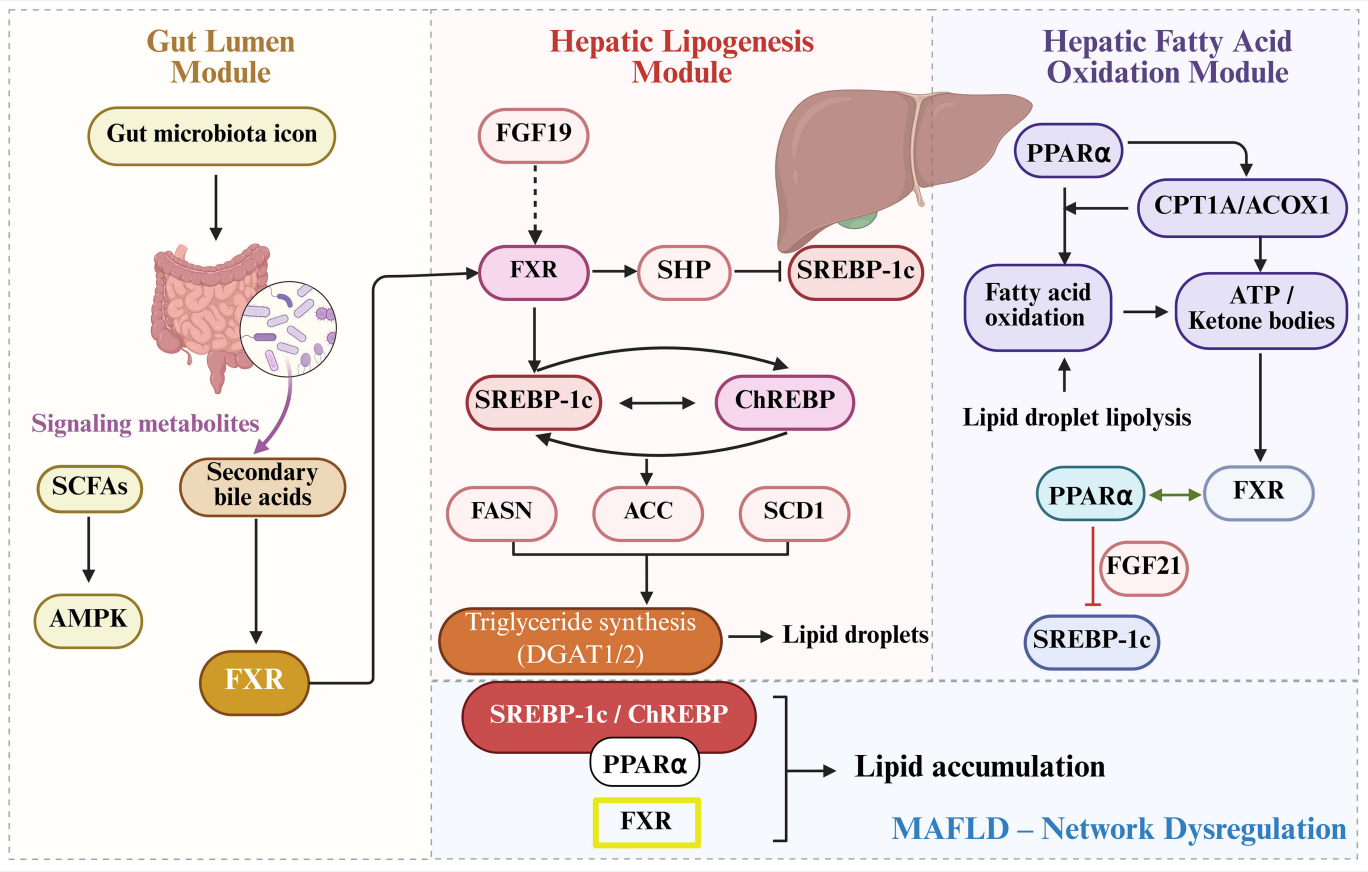
Integrated transcriptional regulatory network governing hepatic lipid metabolism in MAFLD. Schematic of crosstalk among SREBP-1c, ChREBP, PPARα, and FXR in hepatocytes, regulated by gut microbiota-derived metabolites. Arrows indicate activation; T-bars indicate inhibition. Gut microbiota produces secondary bile acids (activating FXR) and SCFAs (modulating AMPK). In the liver, FXR induces SHP to suppress SREBP-1c-mediated lipogenesis, while SREBP-1c and ChREBP synergistically drive *de novo* lipogenesis. PPARα promotes fatty acid oxidation and exhibits bidirectional crosstalk with FXR, while antagonizing SREBP-1c via FGF21. In MAFLD, network dysregulation (SREBP-1c/ChREBP hyperactivation, impaired PPARα and FXR signaling) drives pathological lipid accumulation.

The four transcriptional regulators discussed above—SREBP-1c, ChREBP, PPARα, and FXR—do not operate in isolation. Instead, they form an integrated network that maintains hepatic lipid homeostasis by integrating nutritional, hormonal, and microbial signals (**Figure 3**). Understanding how these factors cooperate and oppose each other is essential for unraveling MAFLD pathogenesis and developing rational therapeutic strategies.

SREBP-1c and ChREBP work together to drive *de novo* lipogenesis. Insulin-activated SREBP-1c and glucose-sensing ChREBP bind cooperatively to promoters of lipogenic genes such as FASN, ACC, and SCD1, ensuring maximal transcriptional output only when both insulin and carbohydrates are abundant. Evidence indicates that both factors are required for the full induction of glycolytic and lipogenic mRNAs after feeding, and they can cooperate epigenetically by promoting histone acetylation at target gene loci. In parallel, PPARα serves as the master regulator of fatty acid oxidation, upregulating CPT1A and ACOX1 in response to fatty acid influx. FXR, activated by bile acids, functions as a central integrator that links bile acid metabolism to lipid homeostasis through both direct hepatic signaling and endocrine crosstalk via intestinal FGF19. These factors engage in intricate crosstalk to maintain metabolic balance. Through induction of the nuclear receptor SHP, FXR creates a negative feedback loop that restrains SREBP-1c-mediated lipogenesis when bile acid levels rise. PPARα and FXR exhibit bidirectional positive regulation: PPARα can upregulate FXR expression, while FXR activation helps maintain bile acid homeostasis, creating conditions that support PPARα-mediated fatty acid oxidation. In contrast, PPARα and SREBP-1c generally oppose each other. PPARα activation promotes fatty acid oxidation and induces FGF21, which systemically suppresses lipogenic pathways, while sustained SREBP-1c activation can indirectly impair PPARα signaling by promoting lipid accumulation that overwhelms oxidative capacity.

The gut microbiota adds another layer of regulation to this hepatic network. Microbiota-derived secondary bile acids such as hyodeoxycholic acid can simultaneously modulate FXR activity and activate PPARα, while short-chain fatty acids influence energy-sensing pathways that affect all four transcriptional regulators. In MAFLD, this network becomes dysregulated at multiple points: persistent activation of SREBP-1c and ChREBP, together with impaired PPARα signaling and disrupted FXR function, collectively drives pathological lipid accumulation. This integrated perspective provides a conceptual framework for multi-target therapeutic strategies aimed at restoring network balance rather than simply inhibiting individual components.

## 5 Potential therapeutic strategies

### 5.1 Lifestyle modification

Lifestyle modification, including regular physical activity (PA) and evidence-based dietary interventions, is widely recognized as the cornerstone of MAFLD and MASH management (107). Exercise plays a critical role in modulating lipid metabolism. An 8-week aerobic training program significantly enhanced hepatic fatty acid β-oxidation through activation of the AMPK-PPARα signaling pathway, thereby alleviating steatosis and inflammation in obese mice (108). Notably, exercise not only upregulated the expression of fatty acid transporter CD36 and desaturase SCD1, promoting lipid metabolic reprogramming, but also improved hepatic TG homeostasis.

Physical activity can reduce serum biomarkers of hepatocellular injury and enhance hepatic TG export and turnover by increasing very-low-density lipoprotein (VLDL) clearance (109). Epidemiological studies have further demonstrated a strong association between sedentary behavior and higher MAFLD prevalence, while experimental data indicate that voluntary wheel running significantly lowers the incidence of HCC in mice (110). From a dietary perspective, the Mediterranean diet (MD) has shown substantial benefits by modulating multiple pathways of lipid metabolism. Unsaturated fatty acids within the MD can remodel hepatic lipid composition, reduce pro-inflammatory lipid accumulation, and increase levels of protective lipid mediators (111, 112). This dietary pattern also decreases liver stiffness measurement (LSM) values and may contribute to a lower risk of HCC (113–116).

A meta-analysis encompassing 26 studies with 3,037 participants confirmed that dietary interventions significantly improve hepatic lipid metabolism. Both calorie-restricted interventions (CRI) and the MD effectively reduce intrahepatic lipid content and correct lipid metabolic disorders (117, 118). Importantly, specific components of the MD may further influence hepatic lipid remodeling by modulating bile acid metabolism and the production of short-chain fatty acids. Collectively, these findings suggest that integrated lifestyle interventions combining exercise and dietary modification can restore metabolic homeostasis through multifaceted regulation of key lipid metabolic pathways and intermediates, providing a robust metabolic foundation for MAFLD prevention and treatment (**Table 3**).

**Table 3:** Therapeutic strategies and interventions targeting MAFLD and MASH.

Category	Drug	Model	Impact on specific lipid species	Effect	Ref.
Lifestyle	PA; Aerobic Training	HFD mice; foz/foz mice	↓ TG, ↓ DAG	PA reduces serum TG, improves whole-body insulin resistance, and decreases hepatic TNF-α, IL-6, and MCP-1 levels. It also reduces p53 expression and inhibits JNK signaling via p27 mediation.	(106–108)
Dietary	Mediterraneandiet (MED)	HFD mice; MAFLD patients	↓ TG	Reduces body weight, improves blood sugar parameters, body fat, Intrahepatic Fat (IHF), and steatosis.	(109–114)
	Intermittent Fasting	HFD mice; MASH patients	↓ TG, ↓ DAG/Cer	Decreased the transition to non-alcoholic steatohepatitis hepatocellular carcinoma (MASH-HCC) and reduced hepatic fat and fibrosis.	(115, 116)
Pharmacological Strategies	Statins	MAFLD Patients; CLDs Patients	↓ FC	Modestly reduce MAFLD patients’ ALT levels, improve MAFLD-related liver histology, reduce hepatic steatosis and fibrosis.	(117, 118)
	PPARα agonist Wy-14,643	MAFLD Patients	↓ TG, ↓ DAG	PPARα agonists suppress anti-fibrotic activity, ballooning, inflammation, and fibrosis.	(119)
	PPAR γ agonists Pioglitazone	MAFLD Patients	↓ TG	PPARγ agonists reduce ballooning, inflammation, and serum aspartate AST levels.	(120)
	FXR agonists Obeticholic acid	MASH patients	↓ TG	Improve MASH, fibrosis, and inflammatory markers.	(121, 122)
Chinese Medicine Monomers and Formulas	Antrodan	HFD mice	↓ TG, ↓ DAG/Cer	Antrodan relieved MAFLD by activating AMPK/SIRT1/SREBP-1c/PPARγ pathway.	(123)
	Curcumin	HFD mice	↓ TG, ↓ DAG	Curcumin improved SLC13A5/ACLY dysregulation through AMPK/mTOR signaling.	(124)
	TF3	OA-induced HepG2 and HEK 293T cells	↓ TG, ↓ lipid droplets	TF3 suppressed lipid deposition through targeting PK/AMPK axis.	(125)
	Shenge Formula	HFD mice	↓ TG, ↓ DAG	SCF inhibits ACOX1 activity, activates PPARα, upregulates CPT1A expression, increases mitochondrial β-oxidation, and reduces lipid accumulation.	(126)
	Xiezhuo Tiaozhi formula	HFD mice	↓ TG, ↓ Cer	Via SIRT1, the formula relieves M1 macrophage polarization and inflammation, inhibiting hepatocyte apoptosis in tissue samples.	(127)

### 5.2 Pharmacological strategies

Pharmacological approaches offer diverse avenues for modulating hepatic lipid metabolism and have opened new perspectives for MAFLD therapy. Statins, as classical lipid-lowering agents, not only improve hepatic histopathology and delay the progression of steatohepatitis and fibrosis, but also significantly reduce cardiovascular events and all-cause mortality in MAFLD patients (119, 128). The latest guidelines from the American Association for the Study of Liver Diseases (AASLD) recommend statins as part of lipid management in MAFLD (120). In receptor-targeted therapies, PPARα deficiency is associated with aggravated hepatic steatosis, whereas selective PPARα agonists such as Wy-14,643 effectively reverse MASH and fibrosis (121). The PPARγ agonist pioglitazone (30 mg/day) has been shown to significantly improve steatosis, inflammation, and hepatocellular ballooning in non-diabetic MASH patients, while also enhancing insulin sensitivity and reducing liver enzyme levels (122). Furthermore, obeticholic acid (OCA), an FXR ligand, has demonstrated histological improvement in MASH and fibrosis in clinical trials, although pruritus remains a notable adverse effect (123, 124). Collectively, these advances highlight the need for individualized, multi-targeted therapeutic strategies to restore metabolic homeostasis in MAFLD.

### 5.3 Traditional Chinese medicine monomers and formulas

Recent research has underscored the remarkable potential of traditional Chinese medicine (TCM) in MAFLD therapy, offering unique advantages through multi-target regulatory mechanisms. Antrodan has been shown to activate the AMPK/Sirt1 signaling pathway, suppressing SREBP-1c and PPARγ expression, thereby ameliorating lipid metabolic disturbances and insulin resistance induced by a high-fat, high-fructose diet (HFD) (125). Curcumin exerts its effects by modulating the SLC13A5/ACLY citrate metabolic axis, inhibiting hepatic citrate transport and metabolism, and correcting lipid deposition through the AMPK–mTOR pathway (126). Theaflavin TF3 directly binds to and inhibits plasma kallikrein (PK), activating AMPK and its downstream cascades to reduce hepatic lipid droplet accumulation (127).

Beyond single compounds, TCM formulas demonstrate integrated regulatory capacity over complex lipid metabolic networks. The Shenge Formula (SGF), a clinically applied TCM prescription for MAFLD, targets inhibition of ACOX1 activity while activating the PPARα–CPT1A pathway to promote mitochondrial β-oxidation, significantly reducing hepatic lipid accumulation (129). The Xiezhuo Tiaozhi Formula (XZTZ), developed specifically for MAFLD, improves the hepatic inflammatory microenvironment by activating the SIRT1 pathway, modulating macrophage polarization, and suppressing pyroptosis (130). Together, these findings highlight the multifaceted therapeutic potential of TCM in MAFLD through coordinated regulation of lipid metabolism, energy balance, and inflammatory signaling, providing new mechanistic insights and complementary strategies for disease intervention.

## 6 Discussion

Hepatic lipid metabolism disorder is a key feature in the development and progression of MAFLD. Intracellular lipid balance is maintained through the coordination of lipid uptake, synthesis, oxidation and secretion. When this balance is disturbed, excess triglycerides accumulate in the liver and lead to steatosis. However, recent studies suggest that lipotoxicity caused by specific toxic lipids, including ceramides, diacylglycerols (DAGs) and free cholesterol (FC), is more closely related to the transition from simple steatosis to MASH, fibrosis and even hepatocellular carcinoma (131). Therefore, eliminating pathogenic lipid species may be more important than simply reducing total liver fat (132).

A major question is whether current therapeutic approaches target the source of lipotoxicity or only improve downstream metabolic changes. We summarize and compare several representative classes of drugs in **Table 4**. FXR agonists such as obeticholic acid (OCA) can inhibit lipogenesis through SHP and promote fatty acid oxidation via interaction with PPARα, thus exerting anti-fibrotic effects (133). However, the widespread transcriptional effects of FXR agonists often lead to side effects such as pruritus and increased LDL cholesterol. In addition, their effects on specific toxic lipids such as ceramides and DAGs are still unclear (134).

**Table 4:** Comparison of representative pharmacological strategies for MAFLD.

Drug Class	Examples	Advantages	Limitations	Impact on Toxic Lipids
FXR agonists	Obeticholic acid (OCA), cilofexor	Antifibrotic; improves bile acid homeostasis	Pruritus, LDL cholesterol elevation; effects on ceramides/DAG unclear	Unknown
PPARα agonists	Pemafibrate, fenofibrate	Improves lipid profiles; favorable safety	Modest fibrosis benefit; no clear reduction of ceramides/DAG	Unclear
PPARγ agonists	Pioglitazone	Insulin sensitization	Weight gain, fluid retention; not fibrosis-specific	Indirect (via reduced lipid flux)
SCD1 inhibitors	Aramchol	Directly targets lipotoxicity	Modest clinical efficacy; compensatory activation of SREBP-1c	Reduces precursors of DAG and ceramides

PPAR agonists have different but complementary functions. PPARα agonists such as pemafibrate enhance fatty acid oxidation and improve lipid profiles, while PPARγ agonists such as pioglitazone improve insulin sensitivity (135). However, neither type of agonist has been clearly shown to reduce hepatic ceramides or DAGs in clinical lipidomic studies (136). PPARγ agonists may also cause weight gain and fluid retention, which may weaken their overall therapeutic benefits. SCD1 inhibitors represented by Aramchol act by reducing the production of monounsaturated fatty acids, thereby limiting the synthesis of DAGs and ceramides (137). Although this mechanism directly targets lipotoxicity, clinical efficacy is relatively limited. One possible reason is the compensatory activation of other lipogenic pathways such as SREBP-1c, which reduces the effect of single-target inhibition (138). Given these limitations, combination therapies that target multiple points in the metabolic network may be more effective. For example, combined activation of FXR and PPARα may synergistically inhibit lipogenesis and enhance oxidation. Similarly, SCD1 inhibitors combined with treatments that promote toxic lipid clearance may help overcome compensatory mechanisms.

Another common challenge is how to specifically target pathogenic lipids without affecting normal physiological lipid functions. Many lipid molecules have subtype- and location-specific functions. For example, C16:0-ceramide promotes lipotoxicity, while C24:0-ceramide may be protective. DAGs located on the cell membrane affect insulin signaling, whereas those stored in lipid droplets are relatively inactive. Most current drugs cannot distinguish between these differences, which may lead to off-target effects. Future drug design should rely more on lipidomics and spatial biology to achieve more precise intervention. The metabolic network also shows strong plasticity. Inhibition of one pathway often activates other pathways as compensation. Targeting the gut microbiota provides an alternative way to regulate bile acid metabolism and FXR signaling, which may reduce systemic side effects and bypass the compensatory mechanisms in the liver (139).

In conclusion, current therapeutic drugs can improve certain manifestations of MAFLD, but they are still insufficient to achieve complete remission. Future research should shift from single-pathway intervention to network-based and lipid-specific strategies. Precise identification of pathogenic lipids, development of selective drugs, rational combination therapy and regulation of the gut-liver axis may together promote the development of more effective treatments for MAFLD.

## 7 Limitations and future prospects

While hepatic lipid metabolic dysregulation has been firmly established as a central mechanism in MAFLD pathogenesis, the complexity of its regulatory network remains incompletely understood. Distinct hepatic cell populations appear to exert specific and coordinated functions in maintaining lipid metabolic homeostasis, yet the precise cellular and molecular mechanisms remain to be fully elucidated. Accumulating evidence suggests that certain toxic lipid species, such as specific ceramide subtypes and oxidized phospholipids, play pivotal roles in driving the transition from simple steatosis to MASH. These pathogenic lipids are believed to originate from dysregulated metabolic pathways and to mediate hepatocellular injury, inflammation, and fibrogenesis through multiple interrelated signaling cascades.

Future research should harness the integration of multidisciplinary technologies to address these questions. Combining single-cell sequencing and spatial transcriptomics can reveal lipid metabolic landscapes across different hepatic cellular compartments, while high-resolution mass spectrometry imaging coupled with dynamic lipidomics can track the distribution and transformation of toxic lipid species *in situ*. Furthermore, gene-editing tools and organoid models can be employed to dissect the functional roles of key regulatory nodes. Together, these advanced approaches will provide mechanistic insights and pave the way for precision prevention and targeted intervention strategies for MAFLD.
