# Supplementary figures and images for: Lipid metabolism-MAFLD crosstalk: mechanisms and therapy
Source: Front Endocrinol (Lausanne). 2026 Mar 18;17:1785178. doi: 10.3389/fendo.2026.1785178 (PMC13038599; doi:10.3389/fendo.2026.1785178)

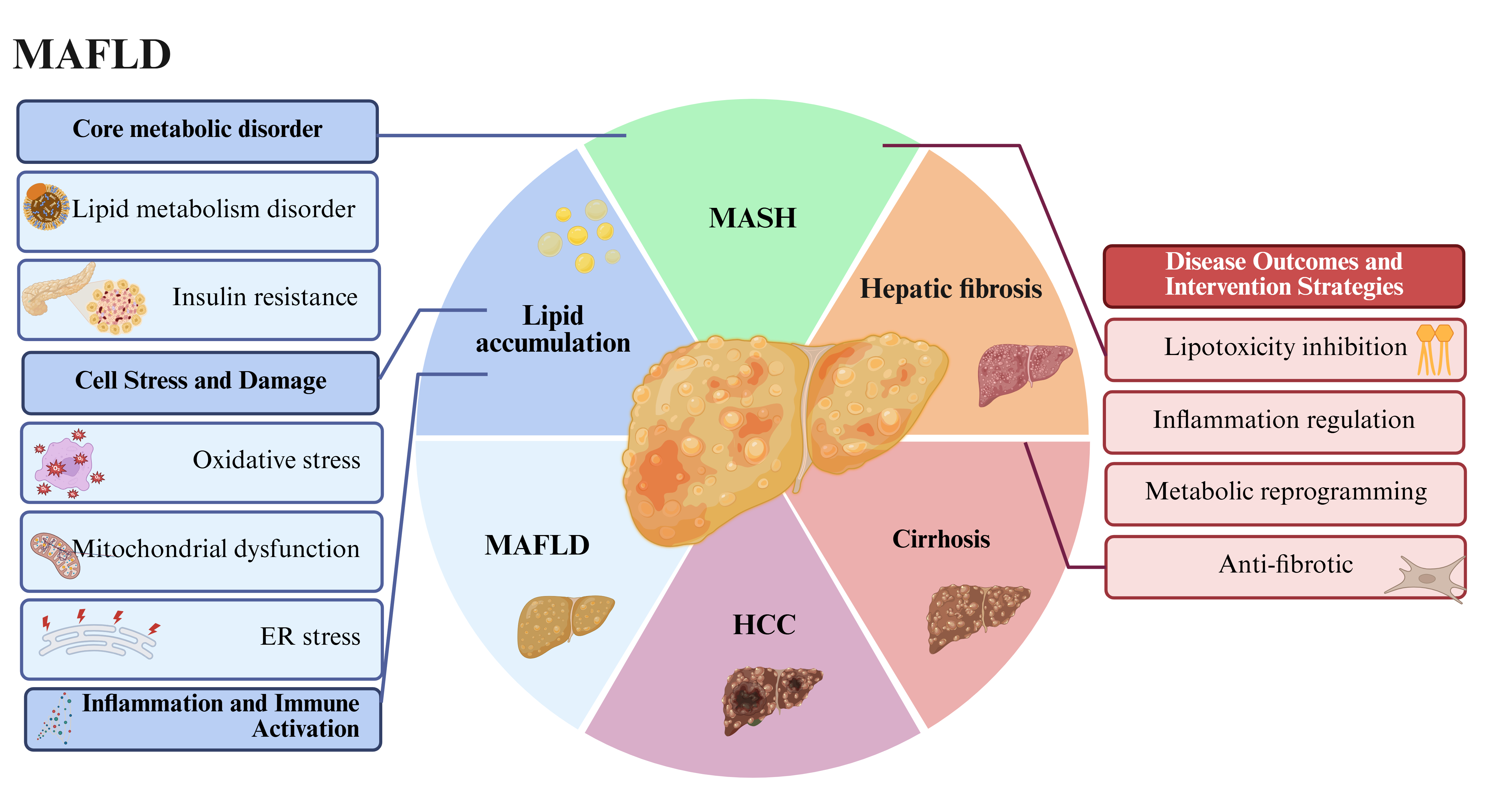

Supplement: Supplementary file 1 [file Image1.jpeg]
